# Differential presence of neutrophil extracellular traps in serum vs plasma of patients with critical limb-threatening ischemia

**DOI:** 10.1016/j.rpth.2026.103441

**Published:** 2026-03-25

**Authors:** Nicolas Gendron, Anne Roche, Vanessa Granger, Joseph Emmerich, Olivier Sanchez, Pascale Gaussem, David M. Smadja, Luc de Chaisemartin

**Affiliations:** 1 Paris Cité University, INSERM, Paris Cardiovascular Research Centre, Team Endotheliopathy and Hemostasis disorders, Paris, France; 2 Hematology Department, Hôpital européen Georges Pompidou, Assistance Publique Hôpitaux de Paris.Centre Université Paris Cité (APHP.CUP), Paris, France; 3 F-CRIN INNOVTE, Saint-Étienne, France; 4 Respiratory Medicine Department, Assistance Publique Hôpitaux de Paris.Centre-Université Paris Cité (APHP.CUP), Paris, France; 5 Assistance Publique Hôpitaux de Paris, Immunology Department, Bichat Hospital, Paris, France; 6 Paris Cité University, INSERM, Center for Research in Inflammation, Team InnaLung, Paris, France; 7 Department of Vascular Medicine, Paris Saint-Joseph Hospital Group, University of Paris, Paris, France; 8 INSERM CRESS UMR, Paris, France

**Keywords:** biomarker, neutrophil extracellular traps, plasma, serum, thrombosis

## Abstract

**Background:**

Neutrophil extracellular traps (NETs) contribute to both arterial and venous thrombosis. However, their detection in peripheral blood remains challenging, with circulating DNA and nucleosomes often used as surrogate markers.

**Objectives:**

This study evaluated circulating NETs, nucleosomes, and double-stranded DNA (dsDNA) in different blood matrices, serum vs plasma, for the quantification of NET markers.

**Methods:**

Thirteen patients with end-stage critical limb-threatening ischemia (CLTI) and 7 controls were included in this retrospective study. NETs (myeloperoxidase [MPO]–DNA complexes), nucleosomes, and dsDNA were quantified in serum and plasma samples from patients with CLTI, and in serum and various plasma types (EDTA, citrate, and heparin) in controls.

**Results:**

MPO–DNA complexes were undetectable in plasma but significantly elevated in the serum of patients with CLTI compared with controls (median optical density, 0.048 vs 0.004; *P* = .012). Serum levels of nucleosomes and dsDNA were also significantly higher in patients with CLTI than in controls (*P* = .029 and *P* = .019, respectively) and higher in serum than in plasma (*P* < .01). In controls, dsDNA and nucleosomes were also higher in serum than in plasma, but MPO–DNA complexes remained undetectable. Correlations among MPO–DNA, nucleosomes, and dsDNA were observed only in serum.

**Conclusion:**

NETs, as evidenced by MPO–DNA complexes, were detectable only in the serum of patients with CLTI, supporting a disease-related origin. Our findings suggest that NET markers are more readily detectable in serum. However, the potential influence of coagulation-related processes during serum preparation should be considered. Our results highlighted the need for methodological standardization in NET biomarker measurements in clinical studies.

## Essentials

• NETs promote arterial and venous thrombosis.
• We compared serum and plasma to measure NET markers in patients with critical limb-threatening ischemia.
• NET markers were detectable in serum but not in plasma samples.
• Blood processing methods may affect NET test results in patients.

## 1 Introduction

Since almost 15 years, increasing literature has pointed out the role of neutrophils in actively supporting and triggering thrombosis, a process now coined immunothrombosis or thromboinflammation [1]. The role of neutrophils in thrombosis comes mainly from their ability to release neutrophil extracellular traps (NETs). NETs consist of extracellular DNA fragments entangled with histones and granular components such as myeloperoxidase (MPO), neutrophil elastase, and cathepsin G. These are released by activated neutrophils through a process known as NETosis, which was initially identified for its antimicrobial properties against bacteria. Recent findings pointed out NETs involvement in many noninfectious diseases such as autoimmune diseases [2,3], diabetes [4], cancer [5,6], atherosclerosis [1], thrombosis [7,8], and, more recently, COVID-19 [9,10]. Von Willebrand factor anchors NETs to the vessel wall, and both von Willebrand factor and NETs recruit platelets, promoting platelet adhesion to the activated endothelium and contributing to thrombosis [11]. The destruction of NETs by addition of DNase or heparin inhibits this phenomenon [11]. Studies revealed the prothrombotic effect of NETs *in vitro* and *in vivo*. NETs were found to promote thrombosis and coagulation [12], in particular thromboinflammatory diseases such as COVID-19 [10], deep venous thrombosis (DVT) [7,13], and stroke [1].

Indeed, NETs can activate coagulation by releasing tissue factor, inhibiting tissue factor pathway inhibitor [12], or activating the contact pathway by binding factor [F]XII [8]. In animal venous thrombosis model, indirect markers of NETs, such as circulating DNA or nucleosomes, are elevated after thrombosis [7,11]. Interestingly, DNase 1 infusion reduce thrombus formation in animal models [7], and mice deficient for PAD4, a nuclear enzyme critical for NETosis, fail to develop DVT [14]. In human, during DVT, circulating nucleosomes [15] and double-stranded DNA (dsDNA) [16] were observed elevated in plasma by comparison with controls. High nucleosome level in plasma was described as an independent risk factor for DVT [15]. Concerning arterial system, NETs have been identified in murine and human atherosclerosis lesions [17]. In stroke, several studies identified elevated levels of dsDNA and nucleosomes [18,19] and provided evidence that extracellular chromatin is a potential therapeutic target [18]. Borissoff et al. [20] showed increased levels of circulating DNA, nucleosomes, and MPO–DNA complexes, considered the most specific marker of circulating NETs, in severe coronary atherosclerosis [20]. MPO–DNA complexes are predictive of the number of atherosclerotic coronary vessels and the occurrence of major adverse cardiac events in this study [20]. The aim of our study was to compare different blood matrices, serum vs plasma, for the quantification of NET markers. To do so, we evaluated the presence of nucleosomes, dsDNA, and circulating NETs measured via MPO–DNA complexes in samples from patients with critical limb-threatening ischemia (CLTI).

## 2 Patients and Methods

This retrospective study included 13 patients that suffered from end-stage CLTI and recruited in the vascular medicine department of the European Georges Pompidou Hospital, Paris, France. Baseline characteristics of the patients with CLTI are presented in Table. Briefly, 12 (92.3%) patients were male, with a median age of 69.1 years (IQR, 59.5-78.6 years) and a median body mass index of 27.4 kg/m^2^ (IQR, 23.9-29.1 kg/m^2^). Most patients presented with cardiovascular risk factors, including diabetes in 6 (46.2%), hypertension in 9 (69.2%), and hyperlipidemia in 9 (69.2%). Two patients (15.4%) were current smokers. Prior to admission, 3 patients (23.1%) had undergone a major amputation, 6 (46.2%) had undergone a minor amputation, and 9 (69.2%) had a history of revascularization. Patients were sampled at the time of admission for management of acute worsening of CLTI. Controls were healthy volunteers of similar age to the patients, with no history of cardiovascular disease (*n* = 7). Informed consent was obtained from all participants (agreement with Paris Descartes University, C CPSL UNT N 12/EFS/064 and OPTIPEC clinical trial, NCT00377897).

**Table:** Baseline characteristics of patients with critical limb-threatening ischemia.

Characteristic	Patients with critical limb-threatening ischemia (*N* = 13)
Demographic data	
Male sex	12 (92.3)
Age (y)	69.1 (59.5–78.6)
Body max index (kg/m^2^)	27.4 (23.9–29.1)
Medical history	
Diabetes	6 (46.2)
Hypertension	9 (69.2)
Hyperlipidemia	9 (69.2)
Current smoking	2 (15.4)
Heart failure	3 (23.1)
Prior stroke	2 (15.4)
Prior myocardial infarction	3 (23.1)
Previous VTE	1 (7.7)
Atrial fibrillation	1 (7.7)
Renal function (mL/min)	
>60	10 (76.9)
30-60	2 (15.4)
<30	3 (23.1)
Previous interventions	
History of major amputation	3 (23.1)
History of minor amputation	6 (46.2)
History of revascularization	9 (69.2)
Antithrombotic	
Aspirin alone	5 (38.5)
Clopidogrel alone	3 (23.1)
Clopidogrel + aspirin	1 (7.7)
Anticoagulant at therapeutic dose	2 (15.4)

Values are given as *n* (%) or median (IQR).

VTE, venous thromboembolism.

Peripheral venous blood samples were collected using BD Vacutainer tubes (Becton Dickinson), including 0.129 M trisodium citrate tubes (citrated plasma, ref: 367714) and nonadditive (serum, ref: 367714) tubes for patients with CLTI, and into citrate, heparin (ref: 368884), EDTA (ref: 368857), and nonadditive tubes for controls (Figure A). Platelet-poor plasma was obtained after a double centrifugation for 15 minutes at 2300*g* and stored at −80 °C until use. Serum was obtained by a single centrifugation for 15 minutes at 2300*g* and stored at −80 °C until use. Circulating NETs (MPO–DNA complexes), nucleosomes, and cell-free dsDNA were measured in both plasma and serum samples. Concentrations of dsDNA in serum and plasma samples were determined by a quantitative fluorimetric assay after DNA extraction and protease digestion. Total DNA was first isolated from 200 μL of sample using the QIAamp UltraSens Virus Kit (Qiagen), and then, DNA concentration was measured using the Quant-iT PicoGreen dsDNA Assay Kit (Life Technologies), according to manufacturer’s instructions. Results were expressed as the DNA concentration in the initial serum or plasma sample in nanograms per milliliter. Circulating nucleosome concentrations were measured by ELISA using the Cell Death Detection ELISA kit (Roche Diagnostics) according to manufacturer’s instructions. Circulating NETs were measured by an in-house ELISA detecting complexes between DNA and MPO, inspired by the technique described by Kessenbrock et al. [21]. Briefly, 20 μL of the samples were mixed with 80 μL of a mixture of biotinylated anti-MPO antibody (dilution 1:50; Abcam ab129370) and horseradish peroxidase–conjugated anti-DNA antibody (dilution 1:25; Cell Death Detection ELISA kit; Roche Diagnostics). The samples were then incubated for 2 hours at room temperature in streptavidin-coated 96-well plates (Roche diagnostics). After 3 washes, the enzyme substrate was added (2,2'-azino-bis [3-ethylbenzthiazoline-6-sulfonic acid]; ABTS; Roche Diagnostics) for 40 minutes at 37 °C, and optical density (OD) was read at 405 nm with a reference wavelength at 492 nm.

**Figure:**
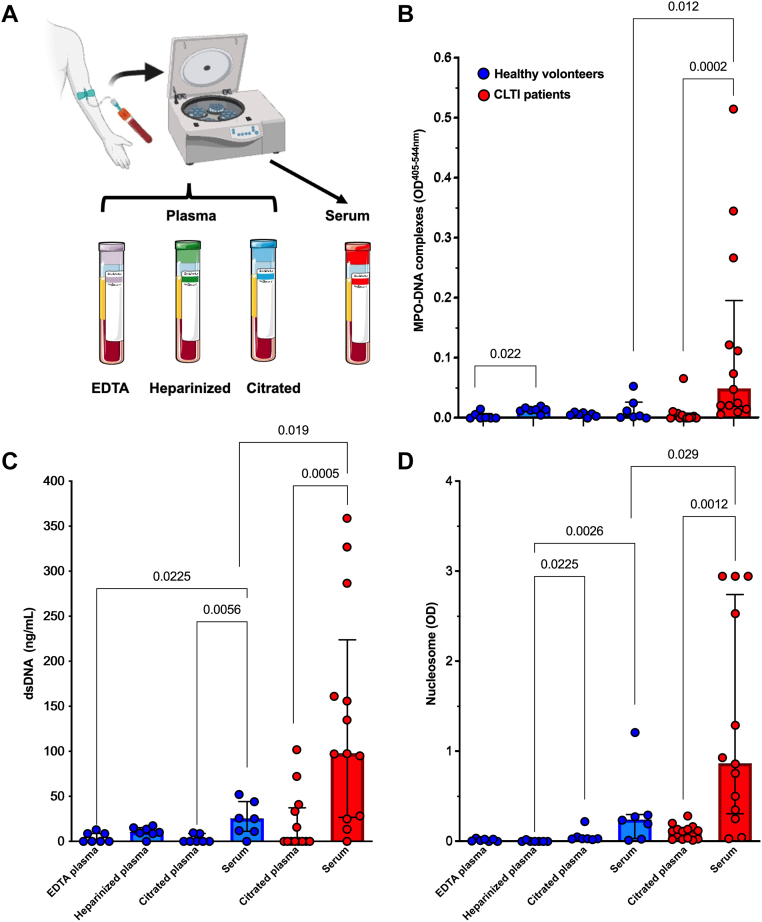
Neutrophil extracellular trap levels in serum and plasma samples in critical limb-threatening ischemia patients and healthy controls. (A) Experimental workflow. (B) Quantification of MPO-DNA complexes. (C) Quantification of dsDNA. (D) Quantification of nucleosomes. Neutrophil extracellular trap biomarker levels in serum and plasma samples in critical limb-threatening ischemia patients (red) and healthy controls (blue). CLTI, critical limb-threatening ischemia; dsDNA, double-stranded DNA; MPO, myeloperoxidase; OD, optical density.

Continuous data were expressed as median (IQR). Data were compared using the Mann–Whitney U-test for continuous variables. For multiple group comparisons, the Kruskal–Wallis test was used for nonparametric variables. Correlation among NET biomarker was assessed using a Spearman correlation test. All analyses were 2-sided, and a *P* value of < .05 was considered statistically significant. Statistical analyses were performed using GraphPad Prism 10.0 software.

## 3 Results and Discussion

First, we observed that median NET levels were low in plasma and serum of controls without difference observed between the samples obtained from the different tubes used (EDTA plasma—OD, 0.000; IQR, 0.000-0.006; citrated plasma—OD, 0.006; IQR, 0.003-0.009; heparinized plasma—OD, 0.014; IQR, 0.012-0.017; serum—OD, 0.004; IQR, 0.001-0.025), except between EDTA plasma and heparinized plasma (*P* = .022) (Figure B). Moreover, median NET levels in citrated plasma of patients with CLTI were also low (OD, 0.001; IQR, 0.000-0.007) and did not significantly differ from controls (*P* = .19). However, median NET levels were significantly higher in serum of patients with CLTI controls (OD, 0.048; IQR, 0.018-0.19; *P* = .012).

These results were confirmed by measuring indirect markers of NETs, ie, nucleosomes and dsDNA (Figure C and D). Median nucleosome levels were significantly higher in serum of patients with CLTI than serum of controls (OD, 0.861 [IQR, 0.301-2.738] vs 0.237 [IQR, 0.026-0.293]; *P* = .029). Interestingly, nucleosome levels were also higher in serum from patients with CLTI than in plasma (median OD, 0.113; IQR, 0.025-0.149; *P* = .0012). Similarly, median circulating dsDNA levels were significantly higher in serum of patients with CLTI than in serum of controls (97.5 ng/mL [IQR, 26.6-223.8 ng/mL] vs 25.1 ng/mL [IQR, 11.0-44.1]; *P* = .019) and higher than in plasma from patients with CLTI (median, 0.0 ng/mL; IQR, 0.0-37.2; *P* = .0005). In controls, we observed that median levels of nucleosomes and dsDNA were low in plasma and serum, but dsDNA levels were significantly higher in serum than citrated and EDTA plasmas (*P* = .0056 and *P* = .0225, respectively), and nucleosome levels were significantly higher in serum and citrated plasma than heparinized plasma (*P* = .0026 and *P* = .0225, respectively). Interestingly, the levels of dsDNA or nucleosome and circulating MPO–DNA complexes were positively correlated only in serum (*r* = 0.63; *P* = .003, and *r* = 0.75; *P* = .0001, respectively) and not in citrated plasma (*r* = − 0.04; *P* = .86 and *r*^2^ = 0.12; *P* = .60, respectively) as previously described in patient with Behcet disease [3].

Previous studies have reported increased levels of surrogate biomarkers of NETs (MPO, dsDNA, and H3Cit) in the plasma of patients with CLTI and described correlations with outcomes such as restenosis and cardiovascular events [22,23]. However, in our study, NETs measured by DNA–MPO complexes were detectable in serum but not in plasma of patients with CLTI. The presence of nucleosome and dsDNA in some type of control plasma in lower concentration than those in serum suggests that coagulation induces a nonspecific DNA release. Nevertheless, the absence of NETs in control serums show that MPO–DNA complexes are linked to patients’ condition and not an artificial coagulation–induced phenomenon. Moreover, NETs did not correlate with neutrophils or white blood cell counts in patients with CLTI (*P* = .505 and *P* = .08, respectively). *In vitro* and animals’ studies suggest the protective effect of DNase [11] and PAD4 inhibitors [24] in NETosis. Such therapies are being developed for human use and should be studied in peripheral arterial disease. To our knowledge, our study is the first to compare NET measurement in plasma of different blood sample origins (EDTA, citrated, and heparinized) and in serum. Borissoff et al. [20] also measured MPO–DNA complexes in plasma of severe coronary atherosclerosis but not in serum. Furthermore, Le Joncour et al. [3] described elevated dsDNA levels and MPO–DNA complexes in serum of patients with Behcet disease than those in controls and more significantly higher levels in patients with vascular involvement of Behcet disease than those in patients without vascular symptoms. Furthermore, Zuo et al. [25] showed that dsDNA levels and MPO–DNA complexes in serum were higher in COVID-19 patients with arterial and venous thrombosis [25]. In citrated plasma, Guy et al. [6] observed higher circulating levels of MPO–DNA complexes but not dsDNA in patients newly diagnosed with myeloproliferative neoplasms who developed thrombosis. In patients with antiphospholipid syndrome, higher dsDNA and MPO–DNA complexes levels were observed in both serum and citrated plasma in comparison with controls [26]. However, comparison between plasma and serum had never been done. So far, studies considering the role of NETs in venous thromboembolism have measured dsDNA in plasma or nucleosomes but not in MPO–DNA complexes to attest the presence of NETs [7,11]. By using MPO and DNA codetection, our ELISA technique allowed us to be more specific of NETs. The reason why NETs were found in serum but not in plasma is unclear aside for heparin tubes since heparin is known to dismantle NETs [11]. In citrated and EDTA plasma, calcium is chelated. Yang and Hayes [27] have shown the role of Ca^2+^ in nucleosome stability. We can thus hypothesize that the chelation of calcium by citrate or EDTA also destabilized NETs, making their detection in plasma impossible. Overall, our hypothesis is that in nonadditive tubes, clot formation upon contact with the tube wall induces platelet activation and coagulation, which in turn triggers the release of NETs [28] in the serum compared with plasma collected in anticoagulated tubes. Thus, the higher levels of NETs observed in patient serum compared with controls could be explained by more NETs being present and more NET-prone neutrophils in patient’s blood due to the thromboinflammatory setting, acting as a 2-hit mechanism.

Our study has several limitations. First, it is a single-center study, including a small sample size of patients. Second, no other NET markers were measured in patient’s samples such as H3Cit [29] or elastase–DNA levels. For example, Rosell et al. [30] showed recently that H3Cit-DNA measured in citrated plasma was an independent predictor for occult cancer in patient after venous thromboembolism. Unpublished data from ongoing projects of the International Society on Thrombosis and Haemostasis Subcommittee on Vascular Biology (2022) indicate that in plasma, MPO–DNA complexes showed good specificity but high variability, H3Cit-DNA demonstrated good specificity with lower variability, and dsDNA lacked specificity and showed high variability. Third, the absence of EDTA and heparin plasma samples from patients with CLTI prevented a full matrix comparison within the patient cohort. Finally, low OD values for MPO–DNA complexes in serum may reflect the use of ABTS, a less sensitive substrate than TMB (3,3',5,5'-tetramethylbenzidine). Our findings suggest that NET markers were more readily detectable in serum than in plasma; however, coagulation-related processes during serum preparation should be considered when interpreting these results. Further studies are required to develop standardized, clinically validated NET markers to correlate NET levels with arterial and venous thrombosis.
